# Legume bioactive compounds: influence of rhizobial inoculation

**DOI:** 10.3934/microbiol.2017.2.267

**Published:** 2017-04-18

**Authors:** Luis R. Silva, Catarina Bento, Ana Carolina Gonçalves, José David Flores-Félix, Martha Helena Ramírez-Bahena, Alvaro Peix, Encarna Velázquez

**Affiliations:** 1 CICS-UBI-Health Sciences Research Centre, University of Beira Interior, Covilhã, Portugal; 2 LEPABE-Department of Chemical Engineering, Faculty of Engineering, University of Porto, Porto, Portugal; 3 Polytechnic Institute of Castelo Branco, Superior School of Health Dr. Lopes Dias, Castelo Branco, Portugal; 4 Departamento de Microbiología y Genética and Instituto Hispanoluso de Investigaciones Agrarias (CIALE), Facultad de Farmacia, Universidad de Salamanca, Salamanca, Spain; 5 Instituto de Recursos Naturales y Agrobiología, IRNASA-CSIC, Salamanca, Spain; 6 Unidad Asociada Universidad de Salamanca-CSIC “Interacción Planta-Microorganismo”, Salamanca, Spain

**Keywords:** legumes, bioactive compounds, rhizobia, inoculation

## Abstract

Legumes consumption has been recognized as beneficial for human health, due to their content in proteins, fiber, minerals and vitamins, and their cultivation as beneficial for sustainable agriculture due to their ability to fix atmospheric nitrogen in symbiosis with soil bacteria known as rhizobia. The inoculation with these baceria induces metabolic changes in the plant, from which the more studied to date are the increases in the nitrogen and protein contents, and has been exploited in agriculture to improve the crop yield of several legumes. Nevertheless, legumes also contain several bioactive compounds such as polysaccharides, bioactive peptides, isoflavones and other phenolic compounds, carotenoids, tocopherols and fatty acids, which makes them functional foods included into the nutraceutical products. Therefore, the study of the effect of the rhizobial inoculation in the legume bioactive compounds content is gaining interest in the last decade. Several works reported that the inoculation of different genera and species of rhizobia in several grain legumes, such as soybean, cowpea, chickpea, faba bean or peanut, produced increases in the antioxidant potential and in the content of some bioactive compounds, such as phenolics, flavonoids, organic acids, proteins and fatty acids. Therefore, the rhizobial inoculation is a good tool to enhance the yield and quality of legumes and further studies on this field will allow us to have plant probiotic bacteria that promote the plant growth of legumes improving their functionality.

## 1. Introduction

The year 2016 was declared by the United Nations as the international year of pulses (grain legumes) recognizing their consumption as beneficial for human health and their cultivation as beneficial for the sustainability of the agriculture due to their ability to fix atmospheric nitrogen (A/RES/68/231) [1]. In this way, the American 2015 Dietary Guidelines recommended the consumption of pulses within a new area named “sustainable diets” [2]. The grain legumes used in human feeding include dry and green beans, broad beans, dry and green peas, chickpeas, lentils, soybeans, lupins, mung beans, and peanuts, among others, and constitute an important source of protein in developing countries [3].

Legumes are a good source of proteins, starch, fibre, vitamins and minerals and also they contain significant quantities of phenolic compounds, such as phenolic acids and flavonoids, which have significant antioxidant properties beneficial to human health [4],[5],[6]. Consumption of legumes reduces the LDL levels and potentially prevents the cardiometabolic risks [7], ischemic heart disease, stroke and type II diabetes [8], hypercholesterolemia [9] and gastrointestinal cancer [10].

One of the most particular characteristics of legumes is their ability to fix atmospheric nitrogen in symbiosis with soil bacteria which are called rhizobia currently distributed in several families and genera [11]. These bacteria induce nodules in legume roots or stems where the nitrogen fixation takes place after the infection process, which included several steps [12]. From colonization to nodule organogenesis different molecules from legumes and bacteria are involved, such as cellulose, cellulases, polysaccharides, lectins, nodulins, flavonoids, etc. [12]. After nodule formation, the rhizobial cells are released into the plant cells and they are transformed into bacteroids which are able to fix atmospheric nitrogen, a process also involving plant and bacterial proteins, such as leghemoglobins and nitrogenases [12].

Therefore, the inoculation of a legume with rhizobia induce metabolic changes in the plant, from which the more studied to date are the increases in the nitrogen and protein contents, and has been exploited in agriculture for improve the crop yield of several legumes [13]. Also, in the last decades the increase of other plant components such as phosporous has been studied after the inoculation of phosphate solubilizing rhizobia [14] and currently the increase of potassium by using K–solubilizing bacteria is starting to be analysed [15]. All these works focused on the analysis of plant components involved in legume yield due to the relevance of biological nitrogen fixation and nutrient mobilization in agriculture in order to reduce chemical inputs allowing health and environment protection [16].

In the last years the study of other legume components whose interest is more related with the human health gained interest. In adition of high quality proteins, legumes contain several bioactive compounds such as polysaccharides, bioactive peptides, phenolics, including isoflavones, carotenoids, tocopherols and fatty acids, amongst other phytochemicals, which make legumes to be excellent functional foods and to include them into the nutraceutical products [6]. Some of these bioactive compounds are influenced by rhizobial inoculation and the aim of this article is to review the state-of-art of research on changes produced in bioactive compounds profiles after rhizobial inoculation of different legumes.

## 2. Rhizobia-legume Symbiosis

Legumes establish nitrogen-fixing symbiosis with a wide variety of soil bacteria collectively called rhizobia, which are currently distributed in several families and genera [11]. All these bacteria are Gram negative aerobic rods with the ability to induce nodules in stems and roots of legumes where, after their transformation into bacteroids, they are able to fix atmospheric nitrogen. The nodules can be formed on roots or on stems and they can be indeterminate with apical meristematic growth or determinate with growth by expansion of infected cells from the central nodule zone [17].

The nitrogen fixation is carried out by rhizobial bacteroids which are the final step of an infection process in which several molecules from both bacteria and plant are involved [12],[18]. This process is mediated by the nitrogenase from the bacteroids which needs a microaerophilic environment facilitated by the leghaemoglobin, a legume protein which removes the oxygen from the symbiosomes and whose expression is observed by the presence of a pink colour in the nodules indicating an effective symbiosis [19].

The rhizobial nitrogen fixation on legumes was the first studied mechanism of plant growth promotion and several studies carried out in field trials showed that rhizobial inoculants can completely replace the chemical fertilization in different legumes increasing their yield and nitrogen content. In this way, the inoculation of the fast growing rhizobial species *Rhizobium leguminosarum* can completely replace the chemical fertilization in *Phaseolus vulgaris* [20]. Also, the inoculation of slow growing strains of *Bradyrhizobium* on *Cajanus cajan* produced the same or even higher yield than the fertilization with mineral nitrogen [21] as well as increases in nitrogen fixation and soil nitrogen uptake by *Pueraria phaseoloides* plants [22].

Therefore, the inoculation with rhizobia is a reliable agronomic practice to increase the production of legumes preserving the environment, which are the main aims of the sustainable agriculture in order to ensure the feeding of the world population in a healthy way.

### 2.1. Legumes with interest for human health

The first interest of legumes is undoubtedly the human nutrition since pulses or grain legumes are one of the most widely consumed foods worldwide due their content in protein, fiber, vitamins and minerals [23]. Legumes have been traditionaly included in the feeding of different cultures and they take part, for example, of the Mediterranean diet [24], where peas, lentils and beans have been traditionally included and whose benefits for human health habe been widely studied [1],[25],[26],[27].

Moreover, several legume seeds contain prebiotic polysaccharides, such as the raffinose family of oligosaccharides, fructooligosaccharides and resistant starch having prebiotic potential [28]–[32]. Also the milk obtained by extrusion of legume seeds and fermented with lactic bacteria, bifidobacteria and/or yeasts can be used as probiotic. This is the case of soybean which has been traditionally consumed in Asiatic countries and currently extended to other continents [33]. In addition to soybean, more recently other pulses have been explored as novel probiotics, such as peanut, lupin, pigeon pea, bambara groundnut, green gram and mung bean [34]–[39].

Nevertheless, in addition to the nutritional value of legumes, several recent studies have focused on the content of legume in bioactive compounds, such as flavonoids, carotenoids, tocopherols, anthocyanes or fatty acids, and their benefits for human health [6],[40].

#### 2.1.1. Bioactive compounds of edible legumes

The most widely known bioactive compounds of legumes are isoflavones which together with phenolic acids and procyanidins constitute the major phenolic compounds present in their seeds [6]. The most studied isoflavones are those from soybean and red clover, whose potential to protect against different diseases, such as cancer, obesity and other metabolic diseases and menopausial symptoms, has been widely reported [6],[41],[42].

Isoflavones are similar to estradiol-17 beta molecules and then they can induce similar effects to those of estrogens but avoiding the risks associated with the treatments with these drugs [43]. These molecules can play a role in cancer diseases related with estrogenic activity, such as breast and endometrial cancer, as suggested recent studies which found a positive effect of soybean intake in woman from Asian and non–Asian countries [44],[45]. Soy foods consumption has also been related with a lower risk of prostate cancer [43],[46] and colorectal cancer [47].

Anthocyanins and proanthocyanidins are phenolic compounds with antioxidant potential present in higher amounts in dark colour seeds than in those of pale colour [48]. Proanthocyanidins (condensed tannins) are present in legume seeds [49]–[52]. All these compounds have antioxidant potential [52],[53] and they can have benefits for human health in the prevention of cardiovascular diseases, diabetes and cancer [54],[55],[56].

Legume seeds also contain different carotenoids (Padhi et al., 2016), being lutein the predominant one in economically important grain legumes, such as soybean, peanut, chickpea, pea, lentil, common and faba bean, cowpea and lupin, followed by zeaxanthin and *β*-carotene [57]. Carotenoids have antioxidant potential [58] and are precursors of vitamin A, which play an important role in age-related macular degeneration and other vision related diseases [59].

Tocopherols comprise compounds with vitamin E activity, being γ-tocopherol the most abundant isoform in lentils, soybean, common bean, pea, chickpea, lentil, broad bean, and some lupin species [57],[58],[60],[61] and α-tocopherol and δ-tocopherol in peanut, cowpea, black-eyed and pinto beans [57],[60]. Vitamin E deficiency may cause neuromuscular problems because it is necessary for the integrity of Purkinje neurons [62] and the uptake of this vitamin is associated with greater fat-free mass [63].

Some legume seeds, such as those of soybean and peanut, constitute an important source of edible oil and several studies have been performed about the fatty acid composition of legume seeds [57],[64]. Unsaturated fatty acids such as oleic and linoleic have been found for example in the seeds of soybean, peanut, lentil, chickpea, beans and lupine [57],[58],[60],[64]. Epidemiological studies showed that a high intake of polyunsaturated fatty acids rather than saturated fatty acids lowers the serum cholesterol [65] and could prevent coronary diseases [66]. Recently has been reported that the replacement of vegetal oils by soybean high-oleic oil and omega-6 polyunsaturated acids has favorable effects on plasma lipid levels reducing cardiovascular risk [67].

Although the number of studies is still low, in the last decade, those analysing the changes in the bioactive compounds content of legumes after rhizobial inoculation are gaining interest. Considering that legumes are currently considered as functional foods taking part of nutraceuticals it is essential to carry out more studies about the differences in the bioactive compound profiles and/or amounts after the inoculation of different legumes with distintic rhizobia.

### 2.2. Rhizobia nodulating edible legumes

Rhizobia are a wide group of bacteria which include species able to establish nitrogen fixing symbiosis with legumes [11]. The two economically most important legumes worldwide, soybean (*Glycine max*) and peanut (*Arachis hypogaea*), are mostly nodulated by different species of the slow-growing genus *Bradyrhizobium* [68]. Nevertheless, soybean can also be nodulated by species of genus *Ensifer*, which was formerly named *Sinorhizobium*, and comprises fast-growing rhizobia [69]. The genus *Bradyrhizobium* is also the main endosymbiont of cowpea (*Vigna unguiculata*) and lupine (*Lupinus* *albus*) [11]. Other worldwide consumed legume is common bean (*Phaseolus vulgaris*), which is nodulated together with pea (*Pisum sativum*), lentil (*Lens culinaris*), faba bean (*Vicia faba*) by fast-growing species from genus *Rhizobium* [70]. Nevertheless, common bean is also nodulated by fast-growing strains of two species from genus *Ensifer*, *Ensifer fredii* and *Ensifer meliloti* [11]. Finally, chickpea (*Cicer arietinum*), which is one of the most cultivated legumes worldwide, is nodulated by several species of genus *Mesorhizobium*, which presented an intermediate growth rate on media containing mannitol as carbon source [71].

The ability of rhizobia to nodulate legumes is linked to the presence of nodulation genes in plasmids, in fast-growing species, or in genomic islands, in slow-growing species and in some intermediate growing species. From these genes, the *nodC* has been used to define symbiovars (previously named biovars) within rhizobial species [11]. The symbiovar concept is related to that of legume promiscuity because there are restrictive legumes that can only be nodulated by a symbiovar such as chickpea or faba bean, and promisuous legumes that can be nodulated by several symbiovars, such as common bean, soybean or cowpea [11]. Legumes such as chickpea, pea, lentil, faba bean and lupine belong to tribes considered restrictive for nodulation, whereas common bean, soybean and cowpea belong to tribes considered as promiscuous for nodulation [72].

In addition to the rhizobia nodulating the most important edible legumes, many others have been described as endosymbionts of these plants [11] and the number of species and symbiovars will increase in the future considering that the vast majority of legumes growing in different ecosystems in the world have not been well studied yet.

## 3. Rhizobial Inoculation and Effect in Legume Bioactive Compounds

The effect of rhizobial inoculation in legumes has been widely studied from an agronomic point of view showing that biofertilization with rhizobia allows to obtain similar legume yields than those obtained with the application of chemical fertilizers [20],[21],[22]. In these studies the parameters commonly analysed are the nodule number and weight, shoot and root weight, grain yield and content in macro and micronutrients, nevertheless in the last decades several studies have been carried about the influence of rhizobial inoculation in the content of bioactive compounds of legumes. One of these studies was carried out on medicinal legumes, such as Psoralea corylifolia L. (Fabaceae), whose seeds are known in traditional Chinese medicine as “Buguzhi” and are widely used for the treatment of various kinds of disorders, particularly vitiligo [73]. The constituents in P. corylifolia L. comprise flavone and coumarin components, such as psoralen [74], a tricyclic furocoumarin with potent photosensitizing property, which is used for the treatment of hypo-pigmented lesions of the skin [75]. It has been reported that the inoculation with *Ensifer meliloti* and *Rhizobium leguminosarum* isolated from *P. corylifolia* nodules in India, increase the psoralen content in the seeds of this legume [76].

Nevertheless, most of studies about the influence of rhizobial inoculation on legume bioactive compounds have been performed in edible legumes highligting those carried out in soybean considering the economic importance of this legume worldwide. Couto et al. [77] showed that the inoculation of *Glycine max* with its common endosymbiont *Bradyrhizobium japonicum* sv glycinearum, in addition of soybean yield, increased the content of phenolic compounds and organic acids in leaves. Also, some volatile compounds such as linalyl acetate, menthyl acetate and α-farnesene only were found in leaves from inoculated soybean plants, which exhibited significantly higher antioxidant activity than those from uninoculated ones. Silva et al. [64] showed that the inoculation with *B. japonicum* sv glycinearum, although does not affect their oxidant capacity, induced changes in the profiles of primary and secondary metabolites of soybean seeds. An increase in some volatile compounds and organic acids was found in seeds from inoculated plants with respect to those from uninoculated ones. Also, inoculated seeds have higher total fatty acids content due to increases in the monounsaturated (MUFA) and polyunsaturated fatty acids (PUFA) contents [64].

Similar results were found after the inoculation of chickpea (*Cicer arietinum*) with a strain of *Mesorhizobium* which does not produced significant increases in antioxidant potential, but significantly increased the content of flavonoids in the seeds [78]. However, a study performed using *Rhizobium leguminosarum* symbiovar viciae as inoculant of faba bean (*Vicia faba*) showed a significant increase in the antioxidant potential of shoots as well as in the content of total phenols, flavonoids, tanins and proteins significantly [79]. Increases in phenolic compounds were also found in shoots and roots of *Arachis hypogaea* after the inoculation of rhizobial strains [80].

In all these studies a single strain of rhizobia was inoculated and the results compared with uninoculated control plants, however many legumes can be nodulated by several symbiovars of rhizobia [11]. In order to analyse the effect of the inoculation of different symbiovars, a study was carried out in cowpea (*Vigna unguiculata*), after the inoculation of *Bradyrhizobium* strains belonging to the symbiovars genistearum and vignae [81]. As occurred with the soybean [64], after the inoculation of bradyrhizobia, the yield of cowpea increased, but particularly when the inoculation was performed using the symbiovar vignae. Similarly, the inoculation decreased the contents of phenol compounds in both soybean and cowpea seeds, although the decrease was lower when cowpea plants were inoculated with the symbiovar vignae [82]. However, whereas the inoculation with *Bradyrhizobium* strains increased the phenolic content of soybean leaves [64], it decreased the phenolic content in cowpea leaves, particularly when the symbiovar genisteae was inoculated in this last legume [82].

Therefore, although further studies by using different rhizobial species and legumes should be carried out, the currently available data showed that the rhizobial inoculation produces changes in the legume bioactive compounds and that a proper selection of the inoculated strains can increase not only the yield of legume crops but also their quality and potential benefits for human health.
